# Polymorphisms of the *XRCC1*, *XRCC3 *and *XPD *genes and risk of colorectal adenoma and carcinoma, in a Norwegian cohort: a case control study

**DOI:** 10.1186/1471-2407-6-67

**Published:** 2006-03-16

**Authors:** Camilla Furu Skjelbred, Mona Sæbø, Håkan Wallin, Bjørn Andersen Nexø, Per Christian Hagen, Inger Marie Bowitz Lothe, Steinar Aase, Egil Johnson, Inger-Lise Hansteen, Ulla Vogel, Elin H Kure

**Affiliations:** 1 Department of Laboratory Medicine, Section of Medical Genetics, Telemark Hospital, N-3710 Skien, Norway; 2 Telemark University College, Faculty of Arts and Sciences, Department of Environmental and Health Studies, Hallvard Eikas plass, N-3800 Bø i Telemark, Norway; 3 National Institute of Occupational Health, Copenhagen, Denmark; 4 Institute of Human Genetics, University of Aarhus, Aarhus, Denmark; 5 Department of Pathology, Ullevaal University Hospital, Oslo, Norway; 6 Department of Pathology, The Gade Institute, Haukeland University Hospital / University of Bergen, N-5021 Bergen, Norway; 7 Department of Gastroenterological Surgery, Ullevaal University Hospital, Oslo, Norway

## Abstract

**Background:**

Genetic polymorphisms in DNA repair genes may influence individual variation in DNA repair capacity, which may be associated with risk of developing cancer. For colorectal cancer the importance of mutations in mismatch repair genes has been extensively documented. Less is known about other DNA repair pathways in colorectal carcinogenesis. In this study we have focused on the *XRCC1*, *XRCC3 *and *XPD *genes, involved in base excision repair, homologous recombinational repair and nucleotide excision repair, respectively.

**Methods:**

We used a case-control study design (157 carcinomas, 983 adenomas and 399 controls) to test the association between five polymorphisms in these DNA repair genes (*XRCC1 *Arg^194^Trp, Arg^280^His, Arg^399^Gln, *XRCC3 *Thr^241^Met and *XPD *Lys^751^Gln), and risk of colorectal adenomas and carcinomas in a Norwegian cohort. Odds ratio (OR) and 95% confidence interval (95% CI) were estimated by binary logistic regression model adjusting for age, gender, cigarette smoking and alcohol consumption.

**Results:**

The *XRCC1 *280His allele was associated with an increased risk of adenomas (OR 2.30, 95% CI 1.19–4.46). The *XRCC1 *399Gln allele was associated with a reduction of risk of high-risk adenomas (OR 0.62, 95% CI 0.41–0.96). Carriers of the variant *XPD *751Gln allele had an increased risk of low-risk adenomas (OR 1.40, 95% CI 1.03–1.89), while no association was found with risk of carcinomas.

**Conclusion:**

Our results suggest an increased risk for advanced colorectal neoplasia in individuals with the *XRCC1 *Arg^280^His polymorphism and a reduced risk associated with the *XRCC1 *Arg^399^Gln polymorphism. Interestingly, individuals with the *XPD *Lys^751^Gln polymorphism had an increased risk of low-risk adenomas. This may suggest a role in regression of adenomas.

## Background

In Western populations the vast majorities of colorectal cancers (CRC) arise from benign adenomatous polyps (colorectal adenomas) and evolve through the adenoma-carcinoma sequence, a multistep process of genetic alterations [1,2]. The lifetime risk of CRC in a normal population is low in comparison to the prevalence of adenomas. This indicates that only a few of the adenomas will develop into a carcinoma. However, from the perspective of understanding the genetic events involved in colonic neoplasia, these early lesions are valuable endpoints.

The risk of sporadic CRC is associated with lifestyle factors like cigarette smoking and alcohol consumption which may be modulated by several genetic factors of low penetrance [3,4]. Cigarette smoke and alcohol may act as sources of chemical carcinogens (including nitrosamines, heterocyclic amines and polycyclic hydrocarbons), reactive oxygen species (ROS) [5,6] and DNA adduct formation [3]. Genetic polymorphisms in DNA repair genes may influence individual variation in DNA repair capacity, which may be associated with risk of developing cancer [7]. During the past years, an increasing number of DNA repair gene polymorphisms have been described and their involvement in carcinogenesis investigated. For colorectal cancer, the importance of mutations in mismatch repair (MMR) genes has been extensively documented. Less is known about other DNA repair pathways in colorectal carcinogenesis, and in this study we have focused on the *XRCC1, XRCC3 *and *XPD *genes.

The DNA repair gene *XRCC1 *gene codes for a protein involved in the repair of single-strand breaks (SSB) and in base excision repair (BER) of damaged bases caused by endogenous and exogenous oxidants. Three polymorphisms occurring at conserved sequences in the *XRCC1 *gene were reported by Shen *et al*. [8]. These coding polymorphisms, resulting in amino acid substitutions, were detected at codons 194 (Arg-Trp), 280 (Arg-His) and 399 (Arg-Gln). Most studies report a reduced risk of cancer associated with the 194Trp allele [9]. The 399 polymorphism have been associated with a number of cancers, although results have been inconsistent [9-15]. Few studies have investigated the association between the *XRCC1 *280His allele and risk of cancer. No association has been observed with colorectal cancer [13], but an increased risk has been reported with lung cancer [16] and breast cancer [17].

The *XRCC3 *gene codes for a protein involved in homologous recombinational repair (HRR) of double-strand DNA and is required for genomic stability [18,19]. The *XRCC3 *gene has a sequence variation in exon 7 (C18067T), which results in an amino acid substitution at codon 241 (Thr^241^Met) that may affect the enzyme's function and/or its interaction with other proteins involved in DNA damage and repair [20]. Molecular epidemiological studies have linked this *XRCC3 *polymorphism to increased risk of breast cancer [21], lung cancer [22], skin cancer [23] and colorectal cancer [14]. The results have been inconsistent [24].

The XPD protein is involved in the nucleotide excision repair (NER) pathway [25], which recognizes and repairs a wide range of structurally unrelated lesions such as bulky adducts and thymidine dimers [26-28]. The *XPD *gene encodes a helicase that is a component of the transcription factor TFIIH [29]. Mutations in the *XPD *gene can diminish the activity of TFIIH complexes increasing the likelihood of repair defects, transcription defects, and abnormal responses to apoptosis [30]. The *XPD *Lys^751^Gln substitution is attributed to a (A→C) transversion at exon 23 [8]. Several epidemiological studies have investigated the association between *XPD *polymorphisms and cancer. Smoking related cancers and skin cancers are most frequently investigated, but it is not yet established whether the polymorphism is linked to risk of lung cancer [31,32]. No association has been observed with the various stages of the adenoma-carcinoma sequence or with colorectal cancer [14,15].

In the present study we used a case-control design to test the association between five amino acid substitution variants of DNA repair genes, *XRCC1 *(Arg^194^Trp), *XRCC1 *(Arg^280^His), *XRCC1 *(Arg^399^Gln), *XRCC3 *(Thr^241^Met) and *XPD *(Lys^751^Gln), and colorectal adenoma/cancer risk in a Norwegian cohort. The main focus was to investigate if these polymorphisms are linked to risk of colorectal adenoma and carcinoma. By studying repair genes in the various stages of the adenoma-carcinoma sequence we may gain new information on the significance of polymorphisms in neoplastic tissues during transition.

## Methods

The cohort in the KAM (Kolorektal cancer, Arv og Miljø) molecular epidemiological study is based on the screening group of the Norwegian Colorectal Cancer Prevention study (The NORCCAP study) in the county of Telemark [33]. The ID number for the NORCCAP study at ClinicalTrials.gov is NCT00119912 [34]. In addition, patients diagnosed with colorectal cancer, operated on at Telemark Hospital (Skien) and Ulleval University Hospital (Oslo) were included. The KAM cohort is based on an ethnic homogeneous group of Norwegian origin.

The KAM biobank consists of blood and tissue samples from 1044 individuals identified with adenomas in the large intestine (991 high- and low-risk adenomas, 53 hyperplastic polyps), 160 with colorectal cancer and 400 controls, defined as individuals with normal findings at flexible sigmoidoscopy screening. All of the participants completed a questionnaire on demographics, health status, dietary; and smoking habits, alcohol consumption, physical exercise and occupation. The questionnaire contained information on a family history of adenomas and carcinomas, and the included cases and controls had no known personal history of genetic predisposition. The KAM study is approved by the Regional Ethics Committee and the Data Inspectorate.

In the present study we analyzed available blood samples from 157 cases with carcinoma, 983 cases with adenomas, (i.e. 227 high-risk and 756 low-risk adenomas) and 399 controls. A high-risk adenoma is defined as an adenoma measuring ≥ 10 mm in diameter and/or with villous components and/or showing severe dysplasia [33].

The distribution of age, gender, cigarette smoking and alcohol consumption among controls and cases with colonic carcinomas and adenomas are shown in Table 1.

Genomic DNA was isolated from blood samples according to standard procedures [35] with minor modifications. In brief, whole blood samples (anticoagulated) was mixed with a threefold volume of lysis buffer (155 mM NH_4_Cl, 10 mM KHCO_3_, 1 mM EDTA pH 7.4) and incubated at 4°C for at least 30 min. The lysate was then centrifuged, and pellet of intact leukocytes was resuspended in 10 ml SE buffer (75 mM NaCl, 24 mM EDTA pH 8.0) 500 μl SDS (20 %) and 50 μl Proteinase K (20 mg/ml) and incubated overnight at 40°C. After digestion, 3.5 ml 6 M NaCl was added to the lysate and the mixture was shaken vigorously and then centrifuged to pellet the cellular proteins. DNA in the supernatant was then precipitated with 2 volumes of absolute ethanol, washed in 70 % ethanol and resuspended in TE buffer (10 mM TrisHCl, 0.1 mM EDTA, pH 7.5).

Genotype analysis of the five single nucleotide polymorphisms (SNPs) of the DNA repair genes *XRCC1*, *XRCC3 *and *XPD *was carried out using the TaqMan allelic discrimination assay on a Sequence Detection System ABI 7000 (Applied Biosystems). The *XRCC1 *polymorphisms (*XRCC1 *Arg^194^Trp (rs#1799782), *XRCC1 *Arg^280^His (rs#25489), *XRCC1 *Arg^399^Gln (rs#25487)) were determined in 12 μl reactions containing 1× MasterMix, 200 nM of each probe, 900 nM primers, and 50–100 ng of genomic DNA. Cycling conditions were as follows: 50°C for 2 min, 95°C for 10 min, and 45 cycles of 95°C for 15 s and 60°C for 1 min. Primers and probes are described in Table 2. The *XRCC3 *Thr^241^Met (rs#861539) and *XPD *Lys^751^Gln (rs#1052559) were genotyped as described previously [24,36]. Controls were included in each run and repeated genotyping of a random 10 % subset yielded 100 % identical genotypes.

SPSS (Statistical Packages for the Social Sciences) 12.0.1 for Windows was used for the statistic calculations. Odds ratios (OR) and 95 % confidence intervals (CI) were calculated using binary logistic regression to assess the relationship between each polymorphism and the colorectal adenoma or carcinoma cases. Two separate ORs were calculated, one adjusted for age only (Table 3), and the other for age, gender, cigarette smoking (number of cigarette years) and alcohol consumption (units of alcohol per month) (Table 4). Due to missing data on cigarette smoking and alcohol consumption (67 controls, 51 carcinomas and 156 adenomas) the number of case and controls used to calculate OR adjusted for age, gender, cigarette smoking and alcohol consumption (in Table 4) is reduced. Possible combined effects of the *XRCC1 *Arg^280^His and *XRCC1 *Arg^399^Gln polymorphisms were investigated in a two by three table (Table 5).

Given the allele frequency of polymorphism of the controls and assuming Hardy-Weinberg equilibrium, we had a 65 % chance (at the 5 % level) of detecting an OR of 2.25 for *XRCC1 *codon 280 heterozygotes among high-risk adenomas and 81 % among low-risk adenomas. We had 60 % chance of detecting an OR of 0.65 among carriers of the variant allele of *XRCC1 *codon 399 among high-risk adenomas and 81 % among low-risk adenomas. For the *XPD *codon 751 polymorphism, we had 54 % chance of detecting an OR of 1.36 and 1.55 among heterozygote and homozygote carriers among high-risk adenomas and 79 % power among low-risk adenomas.

Haplotypes were estimated and ORs calculated using the Hplus program available online [37]. Hplus is a SNP analysis tool for performing haplotype estimation according to the distribution of genotypes in a population. Such haplotype-based methods should be used either for multiple SNPs within candidate genes or when selected SNPs are physically closed to each other [38]. We used this method to estimate haplotypes of three SNPs within the *XRCC1 *gene.

## Results

The genotypic distributions of the five polymorphisms in the three DNA repair genes for both cases and controls are shown in Table 3. The frequencies for the *XRCC1 *194Trp allele, *XRCC1 *280His allele, *XRCC1 *399Gln allele, *XRCC3 *241Met allele and *XPD *751Gln allele among the controls were respectively, 0.06, 0.02, 0.39, 0.40 and 0.34. The genotype distributions were all in Hardy-Weinberg equilibrium and the distributions of the alleles in the control group are in agreement with those found in other Caucasian populations [9], and in other Scandinavian populations for the *XRCC3 *241Met allele and *XPD *751Gln allele [24,36]. Tables 3 and 4 also presents the estimates of ORs of colorectal adenomas and carcinomas associated with the various *XRCC1*, *XRCC3 *and *XPD *polymorphisms.

The *XRCC1 *280His allele was associated with an increased risk of adenomas with OR of 2.30 (95% CI 1.19–4.46) (OR 3.82, 95% CI 1.59–9.18 and OR 2.07, 95 % CI 1.05–4.10 for high- and low-risk adenomas, respectively). Among the carcinoma cases the same trend was observed although not significant. The *XRCC1 *399Gln allele showed a trend towards risk reduction for carcinomas and adenomas, reaching statistical significance only for the high-risk adenomas, OR of 0.62 (95% CI 0.41–0.96). There was no significant association between *XRCC1 *194Trp allele and risk of colorectal adenomas and carcinomas. The combination of the *XRCC1 *codon 280 and *XRCC1 *codon 399 polymorphisms showed a significant positive association for carriers of the *XRCC1 *codon 280 variant allele and *XRCC1 *codon 399 wild type allele, in the high-risk adenomas only, OR of 2.92 (95 % CI 1.20–7.10).

No significant association was found between the *XRCC3 *Thr^241^Met polymorphism and risk of colorectal adenomas and carcinomas.

Further, no significant association was found between the *XPD *Lys^751^Gln polymorphism and colorectal cancer. There was, however, a significant association between the *XPD *751Gln allele and risk of adenomas with OR of 1.42 (95% CI 1.06–1.89). This association was limited to the low-risk adenoma group OR of 1.40 (95% CI 1.03–1.89).

No statistical significant associations were found between the haplotypes estimated in the *XRCC1 *gene and adenoma or carcinoma risk (further data not shown).

## Discussion

In this case-control study we investigated the role of polymorphisms, that result in amino acid substitution, of three DNA repair genes involved in NER, BER and HRR.

In this study we did not find an association between the *XPD *Lys^751^Gln polymorphism and colorectal carcinoma, but we did find an association between the *XPD *751Gln allele and colorectal adenomas, especially with the low-risk adenomas (OR 1.40, 95 % CI 1.03–1.89). Lack of association with the cancer group and the high-risk adenoma group, may be due to small sample size. Another explanation for the observed association in the low-risk adenoma group could be that the polymorphism may play a role in regression of adenomas rather than progression to high-risk adenomas and cancer (advanced neoplasia). Although, it is generally assumed that adenomas grow into cancer or remain in the colon until death, epidemiological data suggests that less than 5 % of adenomas may progress to carcinoma in a lifetime and spontaneous regression of adenomas has been reported [[39-41]]. Hofstad *et al*. [40] have reported that 60 % of adenomas with diameters less than 10 mm stop growing or go into regression within 3 years of observation. In our material, 63 % of cases with low-risk adenomas were hetero- or homozygous for this polymorphism. However, the characteristics of adenomas going into regression are not known.

Our results indicate that the *XPD *Lys^751^Gln polymorphism may predispose slightly to development of colorectal adenomas or that the polymorphism may be of importance for arresting the adenoma-carcinoma sequence in the low-risk phase and facilitate regression of adenomas. Large prospective studies would be required to clarify this issue. *XPD *polymorphisms have been analyzed particularly in epidemiological studies on skin and smoking-related cancers and no obvious relationship has been found for these types of cancers. Mort *et al*. [14] and Yeh *et al*. [15] have reported no significant association between the *XPD *Lys^751^Gln polymorphism and colorectal cancer. To our knowledge no studies have been published on the association between *XPD *polymorphisms and colorectal adenomas.

We found no significant association between the *XRCC1 *194Trp allele and colorectal adenoma or carcinoma risk. This is not in agreement with the results reported by Abdel-Rahman *et al*. [12], but consistent with the results reported by Hong *et al*. [13].

In our study, the *XRCC1 *399Gln allele was associated with a slightly decreased risk of colorectal adenomas and carcinomas, although statistically significant only for the high-risk adenomas, (OR 0.62, 95% CI 0.41–0.96). This protective role of the *XRCC1 *399Gln allele is in contrast to previous reports [12,13] on this BER gene polymorphism and colorectal cancer risk. Previously, we reported a reduced risk of colorectal adenomas and cancer in association with another BER gene polymorphism, the *OGG1 *Ser^326^Cys [42]. We have therefore tested the *XRCC1 *codon 399 and *OGG1 *codon 326 variant alleles in combination, but it showed no significant association with colorectal adenoma or cancer risk (OR 1.18, 95% CI 0.38–3.68; OR 0.91, 95% CI 0.54–1.52) (further data not shown).

In this study we identified an association between the *XRCC1 *280His allele and risk of colorectal adenomas and carcinomas, but again, this increased risk was only significant for the adenomas (OR 2.30, 95% CI 1.19–4.46). As far as we know, the KAM study is the first reporting an association between the *XRCC1 *Arg^280^His polymorphism and colorectal adenoma and carcinoma risk. There are few reports on this polymorphism. In 1999, Lunn *et al*. [43] examined the relationship of *XRCC1 *genotypes and different levels of aflatoxin B1-DNA adducts (51 controls and 69 cases), but no significant effects were observed for the *XRCC1 *280His allele. Recently, Hong *et al*. [13] reported an association between the 280His allele and colorectal cancer in combination with the 194Arg allele and the 399Arg allele (OR 1.78, 95 % CI 1.09–2.89). Takanami *et al*. [44] have reported results suggesting that the *XRCC1 *Arg^280^His variant protein has an ineffective or reduced ability to localize a damaged site in the chromosome, thereby reducing the cellular BER/SSB repair efficiency. The variant 280His allele may allow un-repaired SSBs to accumulate, thereby accelerating genomic instability which consequently increases the risk of carcinogenesis. This is supported by our findings of an association between a variant 280His allele and high-risk adenomas. We found that the two polymorphisms *XRCC1 *Arg280His and *XRCC1 *Arg399Gln were linked such that the variant allele of *XRCC1 *codon 280 co-segregated with the wild type allele of *XRCC1 *codon 399 (p = 0.045). When the *XRCC1 *Arg280His and *XRCC1 *Arg399Gln polymorphisms were combined, the effects of the polymorphisms appeared independently of each other. The variant carriers of *XRCC1 *codon 399 were not statistically significantly, but consistently, at decreased risk of cancer, whereas *XRCC1 *codon 280 heterozygotes were at increased risk of cancer.

No significant association was found between the *XRCC3 *Thr^241^Met polymorphism and colorectal adenomas or carcinomas in our study. This *XRCC3 *codon 241 polymorphism has previously shown a significant association with colorectal cancer risk [14] (OR 1.52, 95% CI 1.04–2.22) while in another study no association was detected [15]. One study reports no association between this *XRCC3 *polymorphism and colorectal adenoma risk [45]. High alcohol consumption has been linked to increased risk of colorectal neoplasia caused by an antifolate effect, resulting in uracil misincorporation into DNA and chromosomal breaks [46].

We have tested the combination of the *XRCC3 *codon 241 variant allele and alcohol consumption but it showed no significant association with colorectal adenoma or carcinoma risk (OR 1.00, 95% CI 0.98–1.02; OR 0.96, 95% CI 0.92–1.00) (further data not shown). We have also tested the interaction between each of the five DNA repair polymorphisms and cigarette smoking, but they showed no significant association with colorectal adenoma or carcinoma (further data not shown). These results are in agreement with other published studies [45,47]. To the best of our knowledge, contrary to genetic factors such as metabolic genetic polymorphisms, few studies suggest that the genetic polymorphisms of DNA repair genes may interact with environmental factors such as alcohol drinking and cigarette smoking [3,9].

The divergence in results from different studies on *XRCC1*, *XRCC3 *and *XPD *polymorphisms may be related to variation in carcinogenic exposure and ethnic origin of the studied populations. Too small sample size and/or the inadequate controlling for certain confounders such as age, gender, alcohol consumption and cigarette smoking may contribute to differing results. It is possible that some of the candidate genes only contribute to colorectal cancer development in combination with certain dietary and /or lifestyle factors. Interaction between various gene products may increase cancer risk. A combination of polymorphisms in these genes may have additive or synergistic effects. For polymorphisms not showing an obvious association with colorectal adenoma or cancer when studied separately, an association with colorectal adenoma or cancer is still possible in combination with other polymorphisms. We have tested the combination of the three polymorphisms in the *XRCC1 *gene but none of the estimated haplotypes showed a significant association with colorectal adenoma or carcinoma risk.

The design of this study is relatively strong because the controls were recruited from the same cohort as the adenoma and carcinoma cases. The CRC cases and controls have not been matched by age which may affect the results in a study. However, our controls have been screened and found polyp free and the risk of any of them having colon cancer at the time of inclusion is not very likely. The definition of polyp-free controls was based on a negative flexible sigmoidoscopy screening examination. It has been estimated that the risk of erroneously classifying individuals with proximal advanced neoplasia as neoplasia-free is less than 3 % at flexible sigmoidoscopy [48,49]. Therefore, the risk of misclassification appears small, and it may only contribute to a type II statistical error, reducing the difference between case groups and controls. It cannot be excluded that the present findings are due to chance. The relatively small numbers of the CRC cases, and the low frequency of the variant allele for at least some of the polymorphisms limited our power to detect an assosiation with risk of adenomas and carcinomas.

## Conclusion

In conclusion, our results suggest an increased risk for advanced colorectal neoplasia in individuals with *XRCC1 *Arg^280^His polymorphism and a reduced risk for persons with *XRCC1 *Arg^399^Gln. The increased risk of having low-risk adenomas in contrast to advanced neoplasia for individuals with *XPD *Lys^751^Gln polymorphism may suggest a role in regression of adenomas and should be explored in prospective studies of *in situ *adenomas.

## Competing interests

The author(s) declare that they have no competing interests.

## Authors' contributions

CFS established the methods in the lab and performed the genotyping analysis of the *XRCC1 *and *XRCC3 *genes and prepared the first draft of the paper. She did the data analysis. MS prepared samples for analysis. She also contributed to the manuscript and with advice on data analysis. PCH contributed with statistical advice. SA and IMBL contributed with the pathology of the cancer cases. EJ participated in the collection of blood samples and questionnaires from the cancer cases. ILH participated in collection and quality control of the questionnaires. UBV contributed with genotyping of the *XPD *gene and together with CFS, MS and EHK interpreted the results. EHK brought the idea of the KAM study and organized it. She was also responsible for the revisions of the manuscript. All authors discussed the results, contributed to interpretation of the results and the final manuscript.

## Pre-publication history

The pre-publication history for this paper can be accessed here:



## Figures and Tables

**Table 1:** Distributions of age, gender, cigarette smoking and alcohol consumption among controls and cases with colorectal carcinomas and adenomas.

	**Controls**	**Carcinomas**	**Adenomas, all**	**High-risk adenomas**	**Low-risk adenomas**
	No (%)	No (%)	No (%)	No (%)	No (%)
**No. of subjects**	399	157	983	227	756
**Gender^a^**					
Male	157 (39.3)	87 (55.4)	603 (61.3)	150 (66.1)	453 (59.9)
Female	242 (60.7)	70 (44.6)	380 (38.7)	77 (33.9)	303 (40.1)
**Age^b^**					
Mean (SD)	54.2 (3.3)	67.9 (11.2)	57.3 (3.7)	57.3 (3.5)	57.3 (3.8)
Smoking status *					
Never smoked	155 (46.7)	33(31.1)	253 (30.6)	51 (27.0)	202 (31.7)
Ever smoked	177 (53.3)	73 (68.9)	574 (69.4)	138 (73.0)	436 (68.3)
Mean no. of cigarette years (SD)	249(229)	383(339)	360(263)	389(244)	351(269)
Alcohol consumption *					
< 1 unit/month	72 (21.7)	36 (34.0)	180 (21.8)	43 (22.8)	137 (21.5)
≥ 1 unit/month	260 (78.3)	70 (66.0)	647 (78.2)	146 (77.2)	501 (78.5)
Mean units of alcohol per month (SD)	11.1 (12.2)	15.5 (22.3)	16.4 (22.9)	18.4 (29.8)	15.8 (20.4)

* Missing values for cigarette smoking and alcohol consumption gave rise to diminished number of cases and controls.

^a ^There are significant differences in the number of males and females in the control group and the case groups, p < 10^-4 ^(test for comparison of two proportions).

^b ^There are significant differences in age among the control group and the case groups, p < 10^-4 ^(Mann-Whitney test).

**Table 2:** Design of primers and TaqMan probes for allelic discrimination assay.

**Primer /Probe**	**Sequence**
*XRCC1 *codon 194	
Forward primer:	5'-GAG GAT GAG AGC GCC AAC TC-3'
Reverse primer:	5'-TCA CTC AGG ACC CAC GTT GTC-3'
Probe C allele:	5'-VIC-TCT TCA GC**C **GGA TCA-MGB/NFQ-3'
Probe T allele:	5'-FAM-TCT TCA GC**T**GGA TCA A-MGB/NFQ-3'
*XRCC1 *codon 280	
Forward primer:	5'-GGT GCT AAC CTA ATC TAC TCT TTG TCT TC-3'
Reverse primer:	5'-TGG GCT CGG TGC CTT CT-3'
Probe G allele:	5'-VIC-AGC TCC AAC TC**G **TAC C-MGB/NFQ-3'
Probe A allele:	5'-FAM-AGC TCC AAC TC**A **TAC C-MGB/NFQ-3'
*XRCC1 *codon 399	
Forward primer:	5'-GAG TGG GTG CTG GAC TGT CA-3'
Reverse primer:	5'-CAT TGC CCA GCA CAG GAT AA-3'
Probe G allele:	5'-VIC-CCT CCC **G**GA GGT AA-MGB/NFQ-3'
Probe A allele:	5'-FAM-CCT CCC **A**GA GGT AAG-MGB/NFQ-3'

The bold underlined letters represent the polymorphic positions.

**Table 3:** Distributions of *XRCC1 *Arg^194^Trp, *XRCC1 *Arg^280^His, *XRCC1 *Arg^399^Gln, *XRCC3 *Thr^241^Met and *XPD *Lys^751^Gln genotypes and development of colorectal carcinomas and adenomas. ORs are adjusted for age.

**Genotypes**	**Controls**	**Carcinomas**	**OR (95% CI)**	**Adenomas**	**OR (95% CI)**	**High-risk adenomas**	**OR (95% CI)**	**Low-risk adenomas**	**OR (95% CI)**
***XRCC1 *Arg^194^Trp**									
Arg/Arg (CC)	352	135	1^a^	864	1^a^	197	1^a^	667	1^a^
Arg/Trp (CT)	46	19	0.99 (0.42–2.33)	115	1.07 (0.72–1.58)	29	1.35 (0.78–2.32)	86	0.99 (0.66–1.50)
Trp/Trp (TT)	1	2	-	4	-	1	-	3	-
Arg/Trp + Trp/Trp	47	21	1.00 (0.43–2.32)	119	1.09 (0.74–1.60)	30	1.38 (0.81–2.36)	89	1.01 (0.68–1.52)
Missing		1							
									
***XRCC1*Arg^280^His**									
Arg/Arg (GG)	381	145	1^a^	906	1^a^	207	1^a^	699	1^a^
Arg/His (GA)	18	12	1.72 (0.58–5.04)	77	1.68 (0.96–2.93)	20	**2.25 **(1.10–4.60)	57	1.55 (0.87–2.76)
His/His (AA)	0	0	-	0	-	0	-	0	-
									
***XRCC1*Arg^399^Gln**									
Arg/Arg (GG)	148	63	1^a^	391	1^a^	100	1^a^	291	1^a^
Arg/Gln (GA)	187	70	0.74 (0.41–1.33)	443	0.82 (0.62–1.08)	95	**0.67 **(0.46–0.99)	348	0.87 (0.65–1.16)
Gln/Gln (AA)	64	24	0.91 (0.42–1.98)	149	0.88 (0.61–1.29)	32	0.65 (0.38–1.13)	117	0.96 (0.65–1.42)
Arg/Gln + Gln/Gln	251	94	0.78 (0.45–1.35)	592	0.84 (0.64–1.08)	127	**0.67 **(0.46–0.96)	465	0.90 (0.68–1.17)
									
***XRCC3 *Thr^241^Met**									
Thr/Thr (CC)	138	64	1^a^	380	1^a^	88	1^a^	292	1^a^
Thr/Met (CT)	201	73	1.15 (0.64–2.08)	456	0.83 (0.63–1.10)	109	0.86 (0.58–1.27)	347	0.81 (0.61–1.09)
Met/Met (TT)	60	20	1.09 (0.46–2.55)	147	0.92 (0.63–1.35)	30	0.84 (0.48–1.47)	117	0.97 (0.65–1.45)
Thr/Met + Met/Met	261	93	1.14 (0.64–2.01)	603	1.11 (0.23–0.85)	139	0.86 (0.59–1.24)	464	0.85 (0.65–1.12)
									
***XPD *Lys^751^Gln**									
Lys/Lys (AA)	175	58	1^a^	364	1^a^	87	1^a^	277	1^a^
Lys/Gln (AC)	173	76	0.92 (0.52–1.63)	463	**1.36 **(1.03–1.78)	100	1.11 (0.75–1.63)	363	**1.43 **(1.07–1.89)
Gln/Gln (CC)	50	22	0.84 (0.36–1.95)	155	**1.55 **(1.05–2.30)	40	1.38 (0.80–2.37)	115	**1.56 **(1.03–2.36)
Lys/Gln + Gln/Gln	223	98	0.90 (0.52–1.54)	618	**1.40 **(1.08–1.81)	140	1.17 (0.81–1.68)	478	**1.46 **(1.11–1.90)
Missing	1	1		1				1	

^a ^The genotype served as reference category

**Table 4:** Distributions of *XRCC1 *Arg^194^Trp, *XRCC1 *Arg^280^His, *XRCC1 *Arg^399^Gln, *XRCC3 *Thr^241^Met and *XPD *Lys^751^Gln genotypes and development of colorectal carcinomas and adenomas. ORs are adjusted for age, gender, cigarette smoking and alcohol consumption.

**Genotypes**	**Controls**	**Carcinomas**	**OR (95% CI)**	**Adenomas**	**OR (95% CI)**	**High-risk adenomas**	**OR (95% CI)**	**Low-risk adenomas**	**OR (95% CI)**
***XRCC1 *Arg^194^Trp**									
Arg/Arg (CC)	292	92	1^a^	734	1^a^	166	1^a^	568	1^a^
Arg/Trp (CT)	39	11	0.82 (0.27–2.47)	89	0.95 (0.61–1.48)	22	1.37 (0.72–2.60)	67	0.85 (0.53–1.36)
Trp/Trp (TT)	1	2	-	4	-	1	-	3	-
Arg/Trp + Trp/Trp	40	13	0.84 (0.28–2.48)	93	0.96 (0.62–1.49)	23	1.35 (0.71–2.56)	70	0.87 (0.55–1.38)
Missing		1							
									
***XRCC1*Arg^280^His**									
Arg/Arg (GG)	319	100	1^a^	764	1^a^	174	1^a^	590	1^a^
Arg/His (GA)	13	6	1.48 (0.29–7.48)	63	**2.30 **(1.19–4.46)	15	**3.82 **(1.59–9.18)	48	**2.07 **(1.05–4.10)
His/His (AA)	0	0	-	0	-	0	-	0	-
									
***XRCC1*Arg^399^Gln**									
Arg/Arg (GG)	119	39	1^a^	326	1^a^	82	1^a^	244	1^a^
Arg/Gln (GA)	159	51	0.87 (0.43–1.78)	371	0.74 (0.54–1.01)	79	**0.59 **(0.37–0.94)	292	0.79 (0.57–1.09)
Gln/Gln (AA)	54	16	1.05 (0.40–2.75)	130	0.93 (0.61–1.42)	28	0.73 (0.39–1.37)	102	0.97 (0.62–1.51)
Arg/Gln + Gln/Gln	213	67	0.91 (0.47–1.79)	500	0.78 (0.59–1.05)	107	**0.62 **(0.41–0.96)	394	0.83 (0.61–1.13)
									
***XRCC3 *Thr^241^Met**									
Thr/Thr (CC)	116	45	1^a^	324	1^a^	70	1^a^	254	1^a^
Thr/Met (CT)	169	48	1.18 (0.57–2.42)	380	0.83 (0.61–1.14)	91	1.06 (0.67–1.68)	289	0.78 (0.57–1.08)
Met/Met (TT)	47	13	1.29 (0.45–3.69)	123	1.03 (0.66–1.60)	28	1.25 (0.65–2.39)	95	1.01 (0.64–1.59)
Thr/Met + Met/Met	216	61	1.20 (0.60–2.39)	502	0.88 (0.65–1.18)	119	1.10 (0.71–1.71)	384	0.83 (0.61–1.13)
									
***XPD *Lys^751^Gln**									
Lys/Lys (AA)	148	43	1^a^	300	1^a^	69	1^a^	231	1^a^
Lys/Gln (AC)	142	47	0.81 (0.40–1.67)	390	1.35 (0.99–1.83)	86	1.27 (0.81–2.01)	304	1.33 (0.97–1.84)
Gln/Gln (CC)	41	15	1.00 (0.38–2.62)	136	**1.66 **(1.07–2.58)	34	1.59 (0.85–2.99)	102	**1.61 **(1.02–2.54)
Lys/Gln + Gln/Gln	183	62	0.86 (0.44–1.68)	526	**1.42 **(1.06–1.89)	120	1.35 (0.88–2.06)	406	**1.40 **(1.03–1.89)
Missing	1	1						1	

^a ^The genotype served as reference category

**Table 5:** Combinations of *XRCC1 *Arg^280^His and *XRCC1 *Arg^399^Gln polymorphisms and risk of colorectal carcinomas and adenomas. ORs are adjusted for age.

**Case group**		**OR (95 % CI) (N _case_/N _controls_)**	
	***XRCC1 *Arg^399^Gln**	***XRCC1 *Arg^280^His**	
**Carcinomas**		GG	GA
	GG	1.0^a ^(56/137)	1.93 (0.44–8.49) (7/11)
	GA	0.76 (0.41–1.38) (65/180)	1.18 (0.20–7.02) (5/7)
	AA	0.96 (0.44–2.09) (24/64)	-
			
**Adenomas**		GG	GA
	GG	1.0^a ^(341/137)	1.75 (0.85–3.63) (50/11)
	GA	0.85 (0.64–1.13) (415/180)	1.22 (0.50–3.00) (27/7)
	AA	0.93 (0.44–1.36) (149/64)	-
			
**High-risk adenomas**		GG	GA
	GG	1.0^a ^(83/137)	**2.92 **(1.20–7.10) (17/11)
	GA	0.77 (0.51–1.15) (92/180)	0.63 (0.14–2.71) (3/7)
	AA	0.74 (0.42–1.29) (32/64)	-
			
**Low-risk adenomas**		GG	GA
	GG	1.0^a ^(258/137)	1.43 (0.66–3.09) (33/11)
	GA	0.88 (0.65–1.18) (324/180)	1.47 (0.59–3.66) (24/7)
	AA	1.00 (0.67–1.48) (117/64)	

^a ^The genotype served as a reference category
